# Case Report: A Rehabilitation Practice Report During ICU Management for a Patient With Multiple Disabilities Due to COVID-19 Pneumonia and COPD

**DOI:** 10.3389/fmed.2021.692898

**Published:** 2021-06-28

**Authors:** Tokio Kinoshita, Ken Kouda, Yasunori Umemoto, Yoshinori Yasuoka, Yuta Minoshima, Yukio Mikami, Yukihide Nishimura, Kyohei Miyamoto, Seiya Kato, Fumihiro Tajima

**Affiliations:** ^1^Department of Rehabilitation Medicine, Wakayama Medical University, Wakayama, Japan; ^2^Division of Rehabilitation, Wakayama Medical University Hospital, Wakayama, Japan; ^3^Department of Rehabilitation Medicine, Iwate Medical University, Iwate, Japan; ^4^Department of Emergency and Critical Care Medicine, Wakayama Medical University, Wakayama, Japan

**Keywords:** SARS-CoV-2, early ambulation, supplemental oxygen therapy, rehabilitation, case report

## Abstract

Patients with severe coronavirus disease (COVID-19) and admitted to the intensive care unit (ICU) are at high risk of developing ICU-acquired weakness and disuse syndrome. Although their medical management may include prolonged deep sedation for pulmonary protection and ventilator management, we aim for early mobilization of these patients with COVID-19. We present the case of a 71-year-old man with chronic obstructive pulmonary disease (COPD) and COVID-19 pneumonia. Passive range of motion training and sitting on the edge of the bed were started in the ICU while the patient was under deep sedation. His activities of daily living eventually improved to where he could independently walk to the toilet without respiratory distress. Patients with severe COVID-19 who require mechanical ventilation are at risk of muscle weakness and exercise intolerance. These patients require rehabilitation therapy, beginning in the acute phase of illness, to recover their physical function. Although validation with a larger cohort is necessary, our results suggest that patients with COPD and COVID-19 pneumonia should undergo rehabilitation concurrently with status-driven changes in respiratory management.

## Introduction

A patient with pneumonia of unknown origin presented in Wuhan, China, in early December 2019 (1). Since that time, a new coronavirus disease (COVID-19) caused by severe acute respiratory syndrome coronavirus 2 (SARS-CoV-2) has spread rapidly throughout the world. As of January 10, 2021, the cumulative number of reported cases worldwide exceeds 80 million, with more than 1.9 million deaths reported (2). The total number of reported cases and in Japan is 339,774, and 4,647, respectively, as of January 20, 2021 (3). Patients with severe COVID-19 who are admitted to an intensive care unit (ICU) are at high risk of developing ICU-acquired weakness (ICU-AW), as well as disuse syndrome. Their medical management often involves prolonged deep sedation for pulmonary protection and ventilator management (4, 5). We also introduce early mobilization to patients with COVID-19 under deep sedation and ventilator management.

In mid-January 2021, a patient with underlying chronic obstructive pulmonary disease (COPD) and pre-morbidly low blood oxygenation was admitted to our hospital with COVID-19 pneumonia. Early rehabilitation therapy was commenced in the ICU, despite the patient being deeply sedated. There have been only a few reports, to date, on rehabilitation for patients with COVID-19 (6–8); however, there have been no reports on rehabilitation during deep sedation for patients with COVID-19 and underlying COPD. In this case report, we provide a practical report delineating information on early rehabilitation for patients with COVID-19 and COPD, the underlying disease, while in the ICU.

## Case Report

The patient was a 71-year-old male with COPD, diabetes mellitus, and a history of percutaneous coronary intervention for unstable angina pectoris. He is currently actively being followed up by the Department of Urology at our hospital following transurethral resection of a bladder tumor for bladder cancer. In mid-January of this year, fatigue and fever reappeared. After 4 days, dyspnea appeared, and worsened 7 days later. Subsequently, he was transported emergently to our hospital and treated with muscle relaxants and sedatives for severe acute respiratory distress syndrome. He was placed on a ventilator and in the prone position under deep sedation for lung protection. Two days after admission, a physiatrist, and a therapist started rehabilitation therapy for expectoration and ICU-AW prevention for severe pneumonia.

At the start of rehabilitation therapy, the patient's respiration was controlled mechanically while under deep sedation (pressure-controlled ventilation, frequency 28, FiO_2_ = 0.5; inspiratory pressure above positive end-expiratory pressure = 12 mmH_2_O; positive end-expiratory pressure = 10 mmH_2_O). The patient was deeply sedated with a Richmond Agitation-Sedation Scale (RASS) score of −5. On the same day, his arterial blood gas values were as follows: pH 7.379; PaO_2_, 98.1 mmHg; PaCO_2_, 61.4 mmHg; PaO_2_/FiO_2_ ratio (P/F), 196; and lactate, 1.1 mmol/L. The patient's Glasgow Coma Scale score was E1VTM1, eyelid conjunctiva was not anemic, and ocular conjunctiva was not yellow. There was no swelling in the cervical lymph nodes. Respiratory sounds were weak with no rales. There was no edema in the extremities but mild range of motion restriction on both shoulders. The patient's laboratory test results were C-reactive protein, 4.49 mg/dL; white blood cells, 1,155/μL; and hemoglobin, 13.0 g/dL.

**Figure 1:**
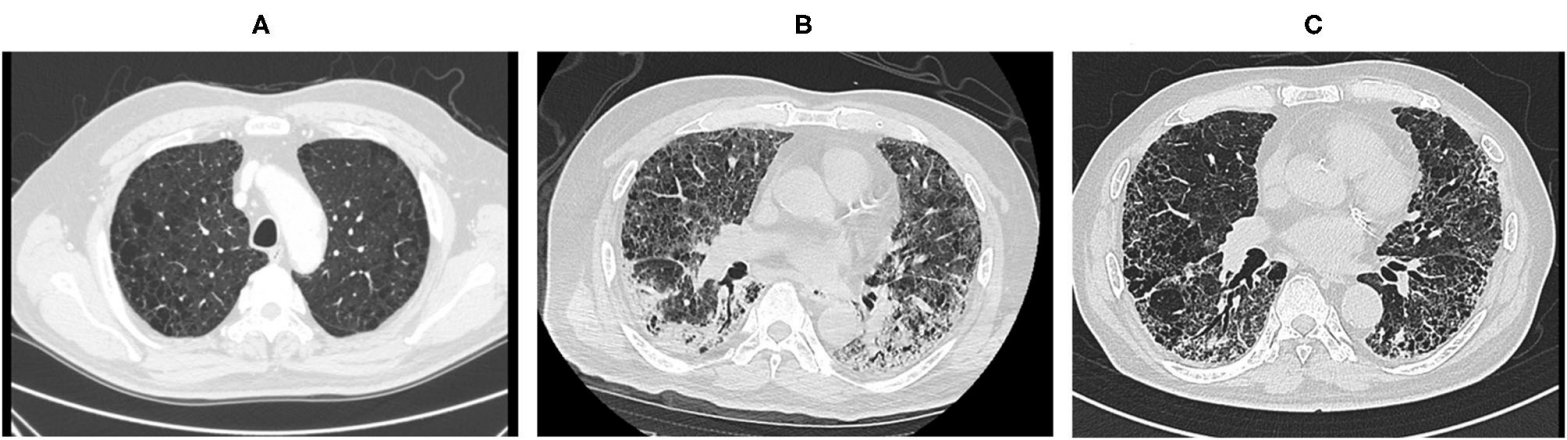
Computed tomography scan before and after hospitalization and before regional hospital transfer. Computed tomography scan images taken **(A)** 1 week prior to hospitalization, **(B)** on hospital day 1, and **(C)** before regional hospital transfer.

The patient was managed in the supine position during the day and prone position at night. His chest computed tomography scan on admission showed an emphysematous lung with diffuse slit-glass shadows and infiltrative shadows on the dorsal side (Figure 1). His Sequential Organ Failure Assessment score (SOFA) was 3 (respiratory = 2, coagulation = 0, hepatic = 0, circulatory = 0, central nervous system = 1, and renal = 0). COPD had not been treated previously, and the pathophysiology before the onset of COPD was unknown. However, a follow-up computed tomography scan taken 1 week before admission revealed significant bilateral emphysematous upper lung field changes. We suspected COPD because of his 50-year smoking history (Figure 1). He was independent for activities of daily living and did not receive home oxygen therapy. However, he continued to experience shortness of breath when walking long distances.

Rehabilitation staff wore personal protective equipment to prevent transmission of COVID-19 by wet biological materials and aerosols from patients during rehabilitation. To reduce infection risk, each patient was treated by only one physical therapist and one nurse.

**Figure 2:**
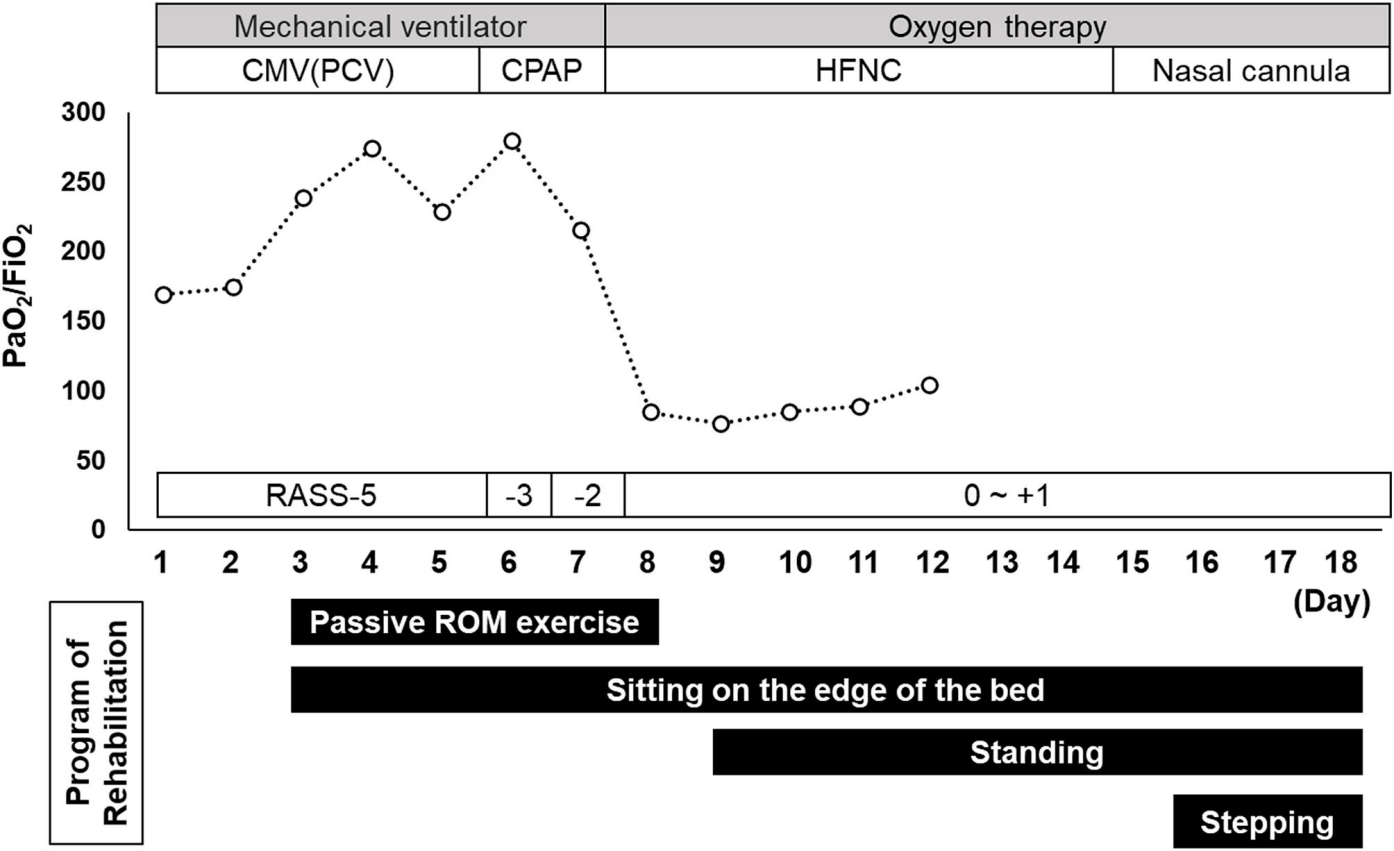
Timeline of the patient's respiratory management and rehabilitation programs. Data recorded in the management of the patient's pulmonary condition included the PaO_2_/FiO_2_ ratio and Sequential Organ Failure Assessment score. Indicated are the rehabilitation programs used between admission (day 1) and the day of regional hospital transfer (day 18). CMV, continuous mandatory ventilation; CPAP, continuous positive airway pressure; HNFC, high-flow nasal cannula; PCV, pressure-controlled ventilation; RASS, Richmond Agitation-Sedation Scale; ROM, range of motion.

**Figure 3:**
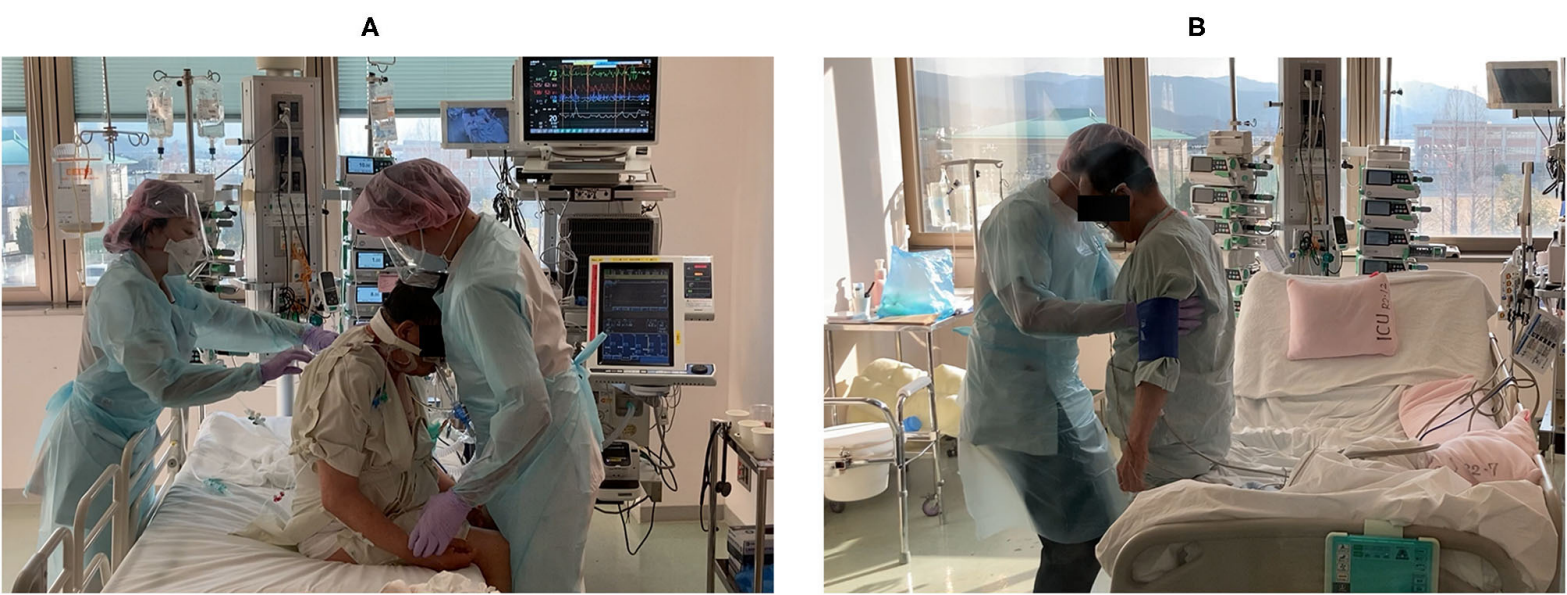
A representative rehabilitation scene. The patient **(A)** sitting on the edge of the bed at the beginning of rehabilitation (day 3) and **(B)** performing half-squats and calf-raises before regional hospital transfer (day 18).

A timeline of the patient's respiratory management and the rehabilitation programs is shown in Figure 2. During the first rehabilitation step, the patient was undergoing RASS-5, deep sedation, and mechanical ventilation; therefore, we were limited to passive range of motion exercises, prevention of ICU-AW and hypovolemia, autonomic adjustment for blood pressure maintenance, and sitting on the edge of the bed to promote expectoration (Figure 3). The patient was placed in a sitting position once a day for at least 20 min by a physical therapist. On hospital day 5 we discontinued the patient's muscle relaxants and tapered the sedative medication. The patient was extubated on hospital day 7 and placed on RASS-2 with a high-flow nasal cannula; 40 L/min and FiO_2_ 80%.

After extubation, the patient's P/F was <100, indicating severely impaired oxygenation, and the saturation percentage of arterial blood oxygen (SpO_2_) at rest during the day ranged from 88 to 92%. He reported no respiratory distress and a pre-morbid computed tomography scan showed emphysematous changes. We therefore determined that he had been nearing hypoxemia before admission. Therefore, the patient's respiratory management and rehabilitation continued.

After transitioning to a high-flow nasal cannula, he could sit independently with his SpO_2_ only decreasing to ~88%. On hospital day 9, he started standing to encourage additional mobilization. Standing training was performed once per day for ~40 min with rest. On hospital day 15, he was transitioned to a standard nasal cannula. At this time, his SpO_2_ dropped to ~80% when standing and he reported feeling short-of-breath. Therefore, we changed to an oxymask, being careful not to cause carbon dioxide narcosis, and the patient was allowed to continue exercising. Next day onward, three sets of exercise were added; these included 50 steps, 20 half-squats, and 20 calf-raises (Figure 3).

By hospital day 18, the patient's muscle strength recovered to the range of 4–5 on the Manual Muscle Test and the patient was able to independently walk to the toilet without respiratory distress. His polymerase chain reaction test for SARS-CoV-2 remained positive and he continued to require oxygen. Consequently, he transferred from our ICU to a regional hospital for continued rehabilitation and preparation for returning home. In this case, no adverse events occurred during rehabilitation until regional hospital transfer. Even after being transferred to the regional hospital, the patient continued strength training and indoor walking training in a private room isolation. Five days after the transfer, the polymerase chain reaction test for SARS-CoV-2 became negative, so isolation was released. The patient needed to be watched over by hospital staffs to prevent falls but was independent in activities of daily living in the hospital. Approximately 1 month after the transfer, the patient was able to walk without supervision, took home oxygen therapy, and returned home. We obtained written informed consent from the patient for the publication of this case report.

## Discussion

There are few reports of rehabilitation for COVID-19 patients in acute care hospitals (8–10), and there have been no reports on acute rehabilitation of patients with COVID-19 and COPD. This is the first report on ICU rehabilitation of a patient with COPD and COVID-19 pneumonia.

In addition to disuse syndrome, patients with COVID-19 are at higher risk of developing ICU-AW (4, 5). For patients managed in the ICU, rehabilitation should be provided as early as possible (11) and a prior report recommended early rehabilitation for patients with COVID-19 (5). A recent review by Goodwin et al. reported that (12) exercise, early mobilization, and multicomponent programs may improve recovery after ICU admission for severe respiratory illness; this may generalize to patients with COVID-19. A review by Bernal-Utrera et al. (13) has revealed that physical therapy for patients with COVID-19 admitted to the ICU is a necessary strategy to prevent complications, contribute to patient stability during critical periods, and promote patient recovery. Wittmer et al. (14) emphasizes the need for early mobilization and exercise for mild, moderate, and severe COVID-19 inpatients to prevent or alleviate illness and ICU-AW. There are still many concerns about the rehabilitation of severe to critical COVID-19 patients; however, some consensus has already been achieved, such as the adoption of breath training in the prone or semi-recumbent bed position, moderate head elevation, limb mobilization, and bed and bedside sitting and standing (15).

The Departments of Emergency and Critical Care and Rehabilitation determined that it was possible to start early mobilization with deeply sedated patients with COVID-19 under ventilator management with muscle relaxants. We therefore commenced sitting on the edge of the bed prior to ventilator weaning. After weaning, the patient's P/F ratio was <100 and his SpO_2_ dropped to ~80% when standing. This made it difficult to continue exercising; however, based on the patient's pre-admission respiratory status, we determined that a SpO_2_ of 88% was acceptable. We thus changed to an oxymask, being careful not to cause carbon dioxide narcosis, and continued the rehabilitation exercises. Liu et al. (16) found that, in patients with COPD, supplemental oxygen during exercise training did not improve exercise capacity, dyspnea score, or quality of life. However, this case demonstrated acute-onset pneumonia caused by COVID-19. With supplemental oxygen, he could leave the bed and start exercising; the authors believed this may have prevented worsening of disuse syndrome. This patient demonstrated early improvements in activities of daily living despite being managed in the ICU and no adverse events occurred during rehabilitation. Therefore, rehabilitation with early mobilization—even for patients dependent on mechanical ventilation—and further status-driven modifications of oxygen therapy may be safe and effective for patients with COPD and COVID-19 pneumonia with multiple disabilities, such as this case.

Older patients and those with underlying medical problems such as hypertension, type II diabetes, cardiovascular disease, chronic lung disease, obesity, and cancer are more vulnerable to severe symptoms due to COVID-19 (17–19). Patients with COVID-19 and COPD are at a higher risk of severe disease and mortality than those without COPD (20). The patient was 71 years old, had a history of COPD and diabetes mellitus, and was at high risk of developing severe symptoms. However, his SOFA score at the start of rehabilitation was 3. His mild vital organ impairment and disease course may have contributed to favorable results.

Patients with COVID-19 admitted to the ICU are known to develop severe fatigue after discharge as a sequela of COVID-19 (21). Ferraro et al. (22) has shown that patient-specific rehabilitation is needed to improve fatigue after transfer from the ICU. Since this case was transferred to a regional hospital, the subsequent symptoms are unknown. In the future, it is necessary to investigate whether early rehabilitation for ICU patients affects the sequelae of COVID-19. Furthermore, since this is a case report of a single patient, the generalizability of the results is not known.

## Conclusions

Patients with severe COVID-19 who require mechanical ventilation are more likely to develop muscle weakness and exercise intolerance; thus, it is important to improve their physical function through early rehabilitation therapy, beginning during the acute disease stage. Although they require validation in a larger cohort, our results suggest that patients with COPD and COVID-19 pneumonia should be rehabilitated concurrently with status-driven changes in respiratory management.

## Data Availability Statement

The original contributions presented in the study are included in the article/Supplementary Material, further inquiries can be directed to the corresponding author/s.

## Ethics Statement

Written informed consent was obtained from the individual(s) for the publication of any potentially identifiable images or data included in this article.

## Author Contributions

KK and TK: concept, idea, and research design. YMik, YN, SK, and FT: consultation (including review of manuscript before submitting). FT: project management. TK: data analysis. TK, YU, YY, and YMin: data collection. KK, TK, and KM: writing. All authors contributed to the article and approved the submitted version.

## Conflict of Interest

The authors declare that the research was conducted in the absence of any commercial or financial relationships that could be construed as a potential conflict of interest.

## References

[1] Huang C Wang Y Li X Ren L Zhao J Hu Y. Clinical features of patients infected with 2019 novel coronavirus in Wuhan, China. Lancet. (2020) 395:497–506. 10.1016/S0140-6736(20)30183-5 31986264 PMC7159299

[2] World Health Organization. Weekly Epidemiological Update on COVID-19. (2021). Available online at: https://www.Weekly_Epidemiological_Update_22.pdf (accessed April 8, 2021).

[3] Ministry of Health Labour and Welfare. COVID-19 Cases in Japan. Available online at: https://www.mhlw.go.jp/stf/newpage_16187.html (accessed April 8, 2021) (in Japanese).

[4] Kress JP Hall JB. ICU-acquired weakness and recovery from critical illness. N Engl J Med. (2014) 370:1626–35. 10.1056/NEJMra1209390 24758618

[5] Thomas P Baldwin C Bissett B Boden I Gosselink R Granger CL. Physiotherapy management for COVID-19 in the acute hospital setting: clinical practice recommendations. J Physiother. (2020) 66:73–82. 10.1016/j.jphys.2020.03.011 32312646 PMC7165238

[6] Curci C Negrini F Ferrillo M Bergonzi R Bonacci E Camozzi DM. Functional outcome after inpatient rehabilitation in post-intensive care unit COVID-19 patients: findings and clinical implications from a real-practice retrospective study. Eur J Phys Rehabil Med. (in press). 10.23736/S1973-9087.20.06660-5 33393278

[7] de Sire A Giray E Ozyemisci Taskiran O. Chelsea physical assessment tool for evaluating functioning in post-intensive care unit COVID-19 patients. J Med Virol. (2021) 93:2620–2. 10.1002/jmv.26867 33570185 PMC8013285

[8] Vlake JH van Bommel J Hellemons ME Wils EJ Gommers D van Genderen ME. Intensive care unit-specific virtual reality for psychological recovery after ICU treatment for COVID-19; a brief case report. Front Med (Lausanne). (2021) 7:629086. 10.3389/fmed.2020.629086 33614677 PMC7892581

[9] Saeki T Ogawa F Chiba R Nonogaki M Uesugi J Takeuchi I. Rehabilitation therapy for a COVID-19 patient who received mechanical ventilation in Japan. Am J Phys Med Rehabil. (2020) 99:873–5. 10.1097/PHM.0000000000001545 32732744 PMC7406209

[10] Nakamura K Nakano H Naraba H Mochizuki M Hashimoto H. Early rehabilitation with dedicated use of belt-type electrical muscle stimulation for severe COVID-19 patients. Crit Care. (2020) 24:342. 10.1186/s13054-020-03080-5 32539827 PMC7294763

[11] Schweickert WD Pohlman MC Pohlman AS Nigos C Pawlik AJ Esbrook CL. Early physical and occupational therapy in mechanically ventilated, critically ill patients: a randomised controlled trial. Lancet. (2009) 373:1874–82. 10.1016/S0140-6736(09)60658-9 19446324 PMC9906655

[12] Goodwin VA Allan L Bethel A Cowley A Cross JL Day J. Rehabilitation to enable recovery from COVID-19: a rapid systematic review. Physiotherapy. (2021) 111:4–22. 10.1016/j.physio.2021.01.007 33637294 PMC7902208

[13] Bernal-Utrera C Anarte-Lazo E Gonzalez-Gerez JJ De-La-Barrera-Aranda E Saavedra-Hernandez M Rodriguez-Blanco C. Could physical therapy interventions be adopted in the management of critically ill patients with COVID-19? A scoping review. Int J Environ Res Public Health. (2021) 18:1627. 10.3390/ijerph18041627 33567748 PMC7915254

[14] Wittmer VL Paro FM Duarte H Capellini VK Barbalho-Moulim MC. Early mobilization and physical exercise in patients with COVID-19: a narrative literature review. Complement Ther Clin Pract. (2021) 43:101364. 10.1016/j.ctcp.2021.101364 33743391 PMC7955568

[15] Li J. Rehabilitation management of patients with COVID-19: lessons learned from the first experience in China. Eur J Phys Rehabil Med. (2020) 56:335–8. 10.23736/S1973-9087.20.06292-9 32329589

[16] Liu Y Gong F. Determination of whether supplemental oxygen therapy is beneficial during exercise training in patients with COPD: a systematic review and meta-analysis. Exp Ther Med. (2019) 18:4081–9. 10.3892/etm.2019.8026 31616520 PMC6781835

[17] Guan WJ Ni ZY Hu Y Liang WH Ou CQ He JX. Clinical characteristics of coronavirus disease 2019 in China. N Engl J Med. (2020) 382:1708–20. 10.1056/NEJMoa2002032 32109013 PMC7092819

[18] Chen N Zhou M Dong X Qu J Gong F Han Y. Epidemiological and Clinical Characteristics of 99 Cases of 2019 novel coronavirus pneumonia in Wuhan, China: a descriptive study. Lancet. (2020) 395:507–13. 10.1016/S0140-6736(20)30211-7 32007143 PMC7135076

[19] Mehra MR Desai SS Kuy S Henry TD Patel AN. Cardiovascular disease, drug therapy, and mortality in COVID-19. N Engl J Med. (2020) 382:e102. 10.1056/NEJMoa2007621 32356626 PMC7206931

[20] Alqahtani JS Oyelade T Aldhahir AM Alghamdi SM Almehmadi M Alqahtani AS. Prevalence, severity and mortality associated with COPD and smoking in patients with COVID-19: a rapid systematic review and meta-analysis. PLoS One. (2020) 15:e0233147. 10.1371/journal.pone.0233147 32392262 PMC7213702

[21] Halpin SJ McIvor C Whyatt G Adams A Harvey O McLean L. Postdischarge symptoms and rehabilitation needs in survivors of COVID-19 infection: a cross-sectional evaluation. J Med Virol. (2021) 93:1013–22. 10.1002/jmv.26368 32729939

[22] Ferraro F Calafiore D Dambruoso F Guidarini S de Sire A. COVID-19 related fatigue: which role for rehabilitation in post-COVID-19 patients? A case series. J Med Virol. (2021) 93:1896–99. 10.1002/jmv.26717 33295637

