# Supplementary material for: Case Report: A Rehabilitation Practice Report During ICU Management for a Patient With Multiple Disabilities Due to COVID-19 Pneumonia and COPD
Source: Front Med (Lausanne). 2021 Jun 28;8:692898. doi: 10.3389/fmed.2021.692898 (PMC8274657; doi:10.3389/fmed.2021.692898)
Supplement: Supplementary file 1 [file Data_Sheet_1.PDF]

CARE case report guidelines

CARE Checklist of information to include when writing a case report

(cc) BY-NC-ND

Topic	Item	Checklist item description	Reported on Line
Title	1	The diagnosis or intervention of primary focus followed by the words "case report"	Line 1-3
Key Words	2	2 to 5 key words that identify diagnoses or interventions in this case report, including "case report"	Line 25, 26
Abstract (no references)	3a	Introduction: What is unique about this case and what does it add to the scientific literature?	Line 29-33
	3b	Main symptoms and/or important clinical findings	Line 34, 35
	3c	The main diagnoses, therapeutic interventions, and outcomes	Line 34-39
	3d	Conclusion—What is the main "take-away" lesson(s) from this case?	Line 39-43
Introduction	4	One or two paragraphs summarizing why this case is unique (**may include references**)	Line 57-61
Patient Information	5a	De-identified patient specific information.	Line 64-67
	5b	Primary concerns and symptoms of the patient.	Line 68-72
	5c	Medical, family, and psycho-social history including relevant genetic information	Line 64-74, 87-93
	5d	Relevant past interventions with outcomes	Line 87-93
Clinical Findings	6	Describe significant physical examination (PE) and important clinical findings.	Line73-93
Timeline	7	Historical and current information from this episode of care organized as a timeline	Figure 2
Diagnostic Assessment	8a	Diagnostic testing (such as PE, laboratory testing, imaging, surveys).	Line 75-93, figure 1
	8b	Diagnostic challenges (such as access to testing, financial, or cultural)	N/A
	8c	Diagnosis (including other diagnoses considered)	Line 70, 71, 87-91
	8d	Prognosis (such as staging in oncology) where applicable	Line 85-87
Therapeutic Intervention	9a	Types of therapeutic intervention (such as pharmacologic, surgical, preventive, self-care)	Line 98-116, figure 2
	9b	Administration of therapeutic intervention (such as dosage, strength, duration)	Line 98-116, figure 2
	9c	Changes in therapeutic intervention (with rationale)	Line 98-116, figure 2
Follow-up and Outcomes	10a	Clinician and patient-assessed outcomes (if available)	Line 117-121
	10b	Important follow-up diagnostic and other test results	Line 122-128
	10c	Intervention adherence and tolerability (How was this assessed?)	Line 104-116
	10d	Adverse and unanticipated events	Line 120,121
Discussion	11a	A scientific discussion of the strengths AND limitations associated with this case report	Line 133-136
	11b	Discussion of the relevant medical literature **with references**.	Line 137-160
	11c	The scientific rationale for any conclusions (including assessment of possible causes)	Line 137-160
	11d	The primary "take-away" lessons of this case report (without references) in a one paragraph conclusion	Line 171-176
Patient Perspective	12	The patient should share their perspective in one to two paragraphs on the treatment(s) they received	N/A
Informed Consent	13	Did the patient give informed consent? Please provide if requested	Yes ☑ No ☐
